# A classification method for neurogenic heterotopic ossification of the hip

**DOI:** 10.1007/s10195-012-0193-z

**Published:** 2012-04-04

**Authors:** Andreas F. Mavrogenis, Giovanni Guerra, Eric Lodwijk Staals, Giuseppe Bianchi, Pietro Ruggieri

**Affiliations:** Department of Orthopaedics, Orthopaedic Oncology Service, Istituto Ortopedico Rizzoli, University of Bologna, Via Di Barbiano 1/10, 40136 Bologna, Italy

**Keywords:** Neurogenic heterotopic ossification, Hip, Brain injury, Spinal cord injury

## Abstract

**Background:**

Existing classifications for heterotopic ossification (HO) do not include all HO types; nor do they consider the anatomy of the involved joint or the neurological injury. Therefore, we performed this study to propose and evaluate a classification according to the location of neurogenic HO and the neurological injury.

**Materials and methods:**

We studied the files of 24 patients/33 hips with brain or spinal cord injury and neurogenic HO of the hip treated with excision, indomethacin, and radiation therapy. We classified patients according to the Brooker classification scheme as well as ours. Four types of neurogenic HO were distinguished according to the anatomical location of HO: type 1, anterior; type 2, posterior; type 3, anteromedial; type 4, circumferential. Subtypes of each type were added based on the neurological injury: a, spinal cord; b, brain injury. Mean follow-up was 2.5 years (1–8 years).

**Results:**

The Brooker classification scheme was misleading—all hips were class III or IV, corresponding to ankylosis, even though only 14 hips had ankylosis. On the other hand, our classification was straightforward and easy to assign in all cases. It corresponded better to the location of the heterotopic bone, and allowed for preoperative planning of the appropriate surgical approach and evaluation of the prognosis; recurrence of neurogenic HO was significantly higher in patients with brain injury (subtype b), while blood loss was higher for patients with anteromedial (type 3) and circumferential (type 4) neurogenic HO.

**Conclusions:**

Our proposed classification may improve the management and evaluation of the prognosis for patients with neurogenic HO.

## Introduction

Heterotopic ossification (HO) is true osteoblastic activity and abnormal formation of mature lamellar bone within extraskeletal soft tissues where bone does not normally exist [1–5]. HO has been classified according to the clinical setting, location of HO, and progressive or isolated occurrence [1–5] into post-traumatic, nontraumatic or neurogenic, and myositis or fibrodysplasia ossificans progressiva [2–11]. Nontraumatic or neurogenic HO or myositis ossificans circumscripta without trauma is frequent in patients with neurological injury; it occurs in 3.4–47 % of patients after spinal cord injury, 10–20 % of patients after closed head injury, and less often after burns, stroke, brain tumors, sickle cell anemia, hemophilia, tetanus, poliomyelitis, multiple sclerosis, and toxic epidermal necrolysis [2, 7, 11]. The incidence is higher in a spastic extremity, patients with complete spinal cord injuries, prolonged immobilization, a high Glasgow coma score, patients in a long coma, and in those with severe spasticity, deep venous thrombosis, hypercalcemia, and hematomas [2, 8, 10, 12, 13]. The most frequent site of neurogenic HO is the hip joint, where it most commonly occurs in the flexor (anterior) or adductor (medial) compartments [2, 8, 10, 11].

There are limited data on the evaluation and management of patients with neurogenic HO [2, 11–14]. Several treatments have been proposed, including surgical excision of the heterotopic bone, radiation therapy, physical therapy, and drugs such as nonsteroidal anti-inflammatory drugs and bisphosphonates [15, 16]. However, most of the data derive from studies on post-traumatic HO, most commonly after total hip arthroplasty [17–19], and most treatments have been based on empirical findings [2, 13]. Additionally, classifications of neurogenic HO are lacking; the classifications that are currently available are related to post-traumatic HO [20–22], and do not address the mechanism of neurological injury or the anatomical compartment involved with the heterotopic bone in order to guide the surgical approach [2–5, 7, 8, 10, 11]. To address these issues, we performed a clinical study of patients with brain and spinal cord injury and neurogenic HO of the hip joint, aiming (1) to propose a classification according to the location of HO at the hip joint and the neurological injury of the patient, and (2) to estimate the prognosis of neurogenic HO based on this classification with respect to range of motion and clinical ankylosis of the hip joint, blood loss and transfusion requirements, and recurrence of the neurogenic HO after combined treatment.

## Materials and methods

We retrospectively studied the medical files of 24 patients with neurogenic HO of the hip joint after central nervous system injury who were diagnosed and treated at the authors’ institution from June 2002 to September 2008. There were 17 male and 7 female patients, with a mean age of 38 years (range, 18–63 years). Sixteen patients had neurogenic HO after brain injury and 8 patients after spinal cord injury; 13 patients were paraplegic, 7 tetraplegic, and 4 hemiplegic. Eighteen of the 24 patients had variable neurogenic HO of the contralateral hip joint; 9 of the 18 patients (patients 4, 9, 10, 12, 15, 18, 19, 21, and 23) also had treatment for neurogenic HO of the contralateral hip at an interval of 6–12 months. Overall, 24 patients/33 hips were included in this study (Table 1). The mean follow-up was 2.5 years (range, 1–8 years); no patient was lost to follow-up. All patients gave written informed consent to be included in this study. This study was approved by the institutional review board/ethics committee of the authors’ institution, and conforms to the latest revision of the Declaration of Helsinki.

**Table 1:** Details of the 24 patients/33 hips included in this study

Patient no.	Gender, age, plegia, treated hip	Brooker classification [1]	Authors’ classification	Follow-up (years)	ROM pre-treatment (°)	ROM post-treatment (°)	Hb pre-treatment (g/dl)	Hb post-treatment (g/dl)	Transfusion (blood units)
1	M, 50, tetraplegia, left hip	Class IV	Type 1a	2	Flexion 10, extension 0; rotation: internal 5, external 5; abduction 0, adduction 0	Flexion 70, extension 20; rotation: internal 30, external 25; abduction 25, adduction 15	15.1	8.9	2
2	M, 45, paraplegia, right hip	Class IV	Type 1a	8	Ankylosis	Flexion 50, extension 10; rotation: internal 15, external 15; abduction 20, adduction 15	12.7	12	–
3	M, 27, tetraplegia, right hip	Class IV	Type 1a	6	Flexion 20, extension 0; rotation: internal 5, external 5; abduction 10, adduction 0	Flexion 80, extension 10; rotation: internal 30, external 25; abduction 25, adduction 20	12.8	10.4	2
4	M, 63, tetraplegia, right hip	Class III	Type 1b	5	Flexion 30, extension 0; rotation: internal 5, external 5; abduction 10, adduction 5	Flexion 90, extension 10; rotation: internal 30, external 30; abduction 30, adduction 25	13.9	10.6	–
	Left hip	Class III	Type 1b	4.5	Flexion 30, extension 0; rotation: internal 5, external 5; abduction 10, adduction 5	Flexion 90, extension 10; rotation: internal 25, external 25; abduction 30, adduction 25	13.9	10.8	–
5	M, 42, paraplegia, right hip	Class III	Type 2a	2	Flexion 40, extension 0; rotation: internal 5, external 10; abduction 0, adduction 0	Flexion 90, extension 20; rotation: internal 15, external 25; abduction 20, adduction 10	12	9.8	–
6	F, 46, paraplegia, left hip	Class IV	Type 2a	1	Ankylosis	Flexion 60, extension 20; rotation: internal 10, external 15; abduction 15, adduction 5	12	9.4	1
7	F, 44, tetraplegia, right hip	Class IV	Type 2b	1	Ankylosis	Flexion 80, extension 15; rotation: internal 10, external 10; abduction 10, adduction 5	13	11	–
8	M, 52, paraplegia, left hip	Class III	Type 2b	1.5	Flexion 30, extension 0; rotation: internal 5, external 5; abduction 0, adduction 0	Flexion 75, extension 15; rotation: internal 15, external 25; abduction 20, adduction 10	11	9.6	–
9	M, 45, tetraplegia, left hip	Class IV	Type 3a	5	Ankylosis	Flexion 80, extension 10 rotation: internal 30, external 30 abduction 30 adduction 20	13.9	9.4	4
	Right hip	Class IV	Type 3a	4	Flexion 30, extension 0 rotation: internal 15, external 20 abduction 20 adduction 10	Flexion 80, extension 10 rotation: internal 30, external 30 abduction 30 adduction 25	14	9	3
10	F, 49, paraplegia, left hip	Class III	Type 3a	2.5	Flexion 30, extension 0 rotation: internal 5, external 5 abduction 10, adduction 0	Flexion 90, extension 20 rotation: internal 30, external 20 abduction 20, adduction 15	11.1	9.5	3
	Right hip	Class III	Type 3a	2	Flexion 10, extension 0 rotation: internal 5, external 5 abduction 5, adduction 0	Flexion 80, extension 20 rotation: internal 25, external 20 abduction 20, adduction 15	15	9.8	3
11	M, 29, paraplegia, right hip	Class IV	Type 3a	1.5	Ankylosis	Flexion 90, extension 20 rotation: internal 30, external 30 abduction 30, adduction 20	13.5	7.5	2
12	M, 35, paraplegia, right hip	Class III	Type 3a	3.5	Flexion 40, extension 5 rotation: internal 10, external 10 abduction 5, adduction 0	Flexion 80, extension 15 rotation: internal 35, external 25 abduction 25, adduction 20	11.5	10.5	–
	Left hip	Class III	Type 3a	3	Flexion 20, extension 5; rotation: internal 10, external 10; abduction 5, adduction 5	Flexion 90, extension 20; rotation: internal 35, external 25; abduction 30, adduction 25	11.6	10.2	–
13	F, 52, tetraplegia, right hip	Class III	Type 3b	4	Flexion 30, extension 0; rotation: internal 5, external 5; abduction 0, adduction 5	Flexion 100, extension 20; rotation: internal 30, external 30; abduction 30, adduction 25	14	12.5	–
14	M, 19, hemiplegia, right hip	Class III	Type 3b	2.5	Flexion 30, extension 30; rotation: internal 5, external 5; abduction 10, adduction 0	Flexion 55, extension 30; rotation: internal 20, external 15; abduction 15, adduction 10	15.2	11.8	2
15	M, 37, paraplegia, right hip	Class IV	Type 3b	1	Flexion 30, extension 30; rotation: internal 0, external 0; abduction 0, adduction 0	Flexion 30, extension 30; rotation: internal 5, external 5; abduction 5, adduction 5	14.3	8.4	4
	Left hip	Class IV	Type 3b	1	Flexion 30, extension 30; rotation: internal 0, external 0; abduction 0, adduction 0	Flexion 30, extension 30; rotation: internal 5, external 5; abduction 5, adduction 5	14.6	9.2	3
16	F, 52, hemiplegia, left hip	Class III	Type 3b	1.5	Flexion 30, extension 0; rotation: internal 5, external 5; abduction 10, adduction 5	Flexion 80, extension 15; rotation: internal 20, external 20; abduction 20, adduction 10	10.9	10	–
17	F, 21, hemiplegia, left hip	Class III	Type 3b	1.5	Flexion 30, extension 0; rotation: internal 10, external 15; abduction 10, adduction 5	Flexion 90, extension 20; rotation: internal 30, external 30; abduction 30, adduction 20	11.5	10.5	–
18	M, 46, paraplegia, right hip	Class IV	Type 3b	4	Ankylosis	Flexion 50, extension 10; rotation: internal 20, external 15; abduction 15 adduction 5	12.4	11	1
	Left hip	Class IV	Type 3b	3	Flexion 50, extension 0; rotation: internal 10, external 5; abduction 5 adduction 5	Flexion 70, extension 10; rotation: internal 20, external 10; abduction 15 adduction 10	12	9	1
19	F, 38, hemiplegia, left hip	Class III	Type 3b	2.5	Flexion 45, extension 0; rotation: internal 10, external 15; abduction 15, adduction 5	Flexion 90, extension 10; rotation: internal 35, external 30; abduction 30, adduction 20	12	7.6	2
	Right hip	Class III	Type 3b	2.5	Flexion 80, extension 0; rotation: internal 15, external 15; abduction 15, adduction 15	Flexion 100, extension 10; rotation: internal 35, external 30; abduction 30, adduction 20	10.7	9.3	1
20	M, 42, paraplegia, right hip	Class IV	Type 3b	3.5	Flexion 20, extension 0; rotation: internal 5, external 5; abduction 10, adduction 10	Flexion 90, extension 20; rotation: internal 30, external 25; abduction 25, adduction 20	14.6	9.2	3
21	M, 30, paraplegia, right hip	Class IV	Type 4a	3.5	Ankylosis	Flexion 90, extension 20; rotation: internal 35, external 30; abduction 35, adduction 25	15.2	11.3	3
	Left hip	Class IV	Type 4a	3	Flexion 40, extension 5; rotation: internal 15, external 10; abduction 20, adduction 15	Flexion 80, extension 20; rotation: internal 30, external 30; abduction 30, adduction 25	12.5	9.5	3
22	M, 30, paraplegia, right hip	Class III	Type 4a	3	Flexion 80, extension 0; rotation: internal 10, external 15; abduction 20, adduction 15	Flexion 100, extension 20; rotation: internal 35, external 30; abduction 40, adduction 25	14.1	9.9	2
23	M, 43, paraplegia, left hip	Class III	Type 4a	3	Flexion 30, extension 0; rotation: internal 5, external 5; abduction 5, adduction 0	Flexion 90, extension 20; rotation: internal 25, external 25; abduction 30, adduction 25	11.3	9.4	3
	Right hip	Class III	Type 4a	2.5	Flexion 10, extension 0; rotation: internal 5, external 5; abduction 5, adduction 0	Flexion 90, extension 20; rotation: internal 20, external 20; abduction 20, adduction 20	13.5	7.5	3
24	M, 24, tetraplegia, left hip	Class IV	Type 4b	6	Flexion 10, extension 0; rotation: internal 0, external 0; abduction 0, adduction 0	Flexion 60, extension 10; rotation: internal 20, external 15; abduction 30, adduction 15	14.1	12.6	–
Summary (mean value, range)					Flexion 25 (0–80), extension 2.5 (0–30); rotation: internal 5.5 (0–15), external 6 (0–20); abduction 6 (0–20), adduction 3 (0–15)	Flexion 79 (30–100), extension 17 (10–30); rotation: internal 25 (5–35), external 23 (5–30); abduction 25 (5–40), adduction 17 (5–25)	13 (10.7–15.2)	9.9 (7.5–12.6)	1.5 (0–4)

*Pts* patients, *ROM* range of motion, *Hb* hemoglobin

Neurogenic HO was classified according to the Brooker classification [1] for post-traumatic HO after total hip arthroplasty (Table 2) as well as the classification proposed herein (Table 3). Our classification is based on the (1) anatomical location of the heterotopic bone as shown in axial computed tomography (CT) scans of the hip and proximal femur, (2) clinical ankylosis of the hip joint, and (3) the etiology of the neurological injury (brain or spinal cord injury). All imaging studies were reviewed by the authors and two radiologists who were asked to classify neurogenic HO according to these two classifications on a consensus basis.

**Table 2:** Brooker classification of HO of the hip

Class	Patients (*n* = 24)/hips (*n* = 33)	Description
I	–	Bone islands within soft tissues about the hip
II	–	Bone spurs in pelvis or proximal end of femur, leaving at least 1 cm between the opposing bone surfaces
III	12/17	Bone spurs that extend from the pelvis or the proximal end of the femur, which reduce the space between the opposing bone surfaces to less than 1 cm
IV	12/16	Radiographic ankylosis of the hip

**Table 3:** Authors’ classification of neurogenic HO of the hip

Type	Patients (*n* = 24)/hips (*n* = 33)	Description
Type 1		
a: Spinal cord injury	3/3	Neurogenic HO at the anterior hip or the proximal end of the femur, with or without ankylosis
b: Brain injury	1/2	
Type 2		
a: Spinal cord injury	2/2	Neurogenic HO at the posterior hip or the proximal end of the femur, with or without ankylosis
b: Brain injury	2/2	
Type 3		
a: Spinal cord injury	4/7	Neurogenic HO at the anterior and medial hip or the proximal end of the femur, with or without ankylosis
b: Brain injury	8/11	
Type 4		
a: Spinal cord injury	3/5	Neurogenic HO around the hip (circumferential), with or without ankylosis
b: Brain injury	1/1	

In all patients, the pre-treatment evaluation included clinical evaluation of the range of motion (flexion, extension, rotation, abduction, and adduction) and ankylosis of the respective hip joint, serial serum alkaline phosphatase measurements and a preoperative measurement of serum hemoglobin, standard radiographs, and at least two three-phase technetium-99 m (99mTc) methylene diphosphonate bone scans to evaluate the maturation of HO. Treatment was applied at a mean of 1 year (range, 0.5–7 years) after the initial imaging evidence of HO to allow for the maturation of HO and facilitate resection with minimum trauma to the surrounding tissue [2]. In all 24 patients/33 hips, treatment included surgical excision of the heterotopic bone followed by radiation therapy in a single fraction of 600 cGy administered within 72 h postoperatively (range, 48–72 h), and indomethacin administration in daily doses of 50–100 mg starting from the first postoperative day for 6 weeks. Postoperatively, blood transfusion requirements were recorded and serum hemoglobin was measured. Post-treatment evaluation, including clinical examination of range of motion and imaging evaluation of the respective hip joint using radiographs and CT scans, was performed at 6-month intervals to evaluate the effect of treatment and the evidence for recurrence of HO. Recurrent neurogenic HO was defined as a reduction in the range of motion obtained after surgery and imaging evidence of HO.

Statistical analysis was performed using Student’s *t* test and the chi-square test. The data were recorded in a Microsoft^®^ Excel^®^ 2003 spreadsheet (Microsoft Corporation, Redmond, WA, USA) and analyzed using MedCalc^®^ software, version 11.1 (MedCalc Software, Mariakerke, Belgium).

## Results

**Fig. 1:**
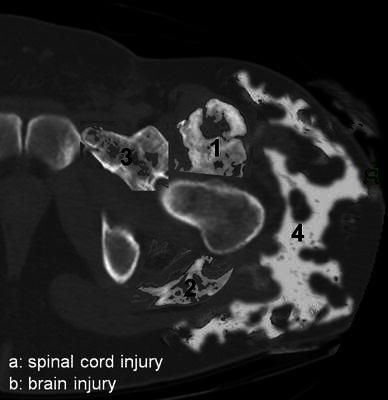
A classification method for neurogenic HO of the hip according to the anatomical location of HO (types *1*–*4*) and the neurological injury (subtypes *a* and *b*)

**Fig. 2:**
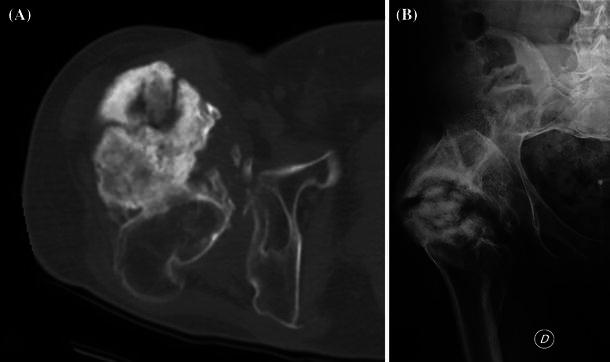
**a** Axial computed tomography scan and **b** anteroposterior radiograph of the right hip of a 63-year-old man with anterior neurogenic HO of the hip after closed brain injury (patient 4; type 1b)

**Fig. 3:**
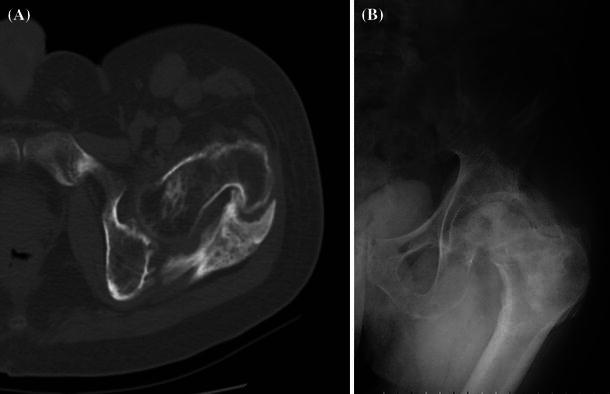
**a** Axial computed tomography scan and **b** anteroposterior radiograph of the left hip of a 52-year-old man with posterior neurogenic HO of the hip after closed brain injury (patient 8; type 2b)

**Fig. 4:**
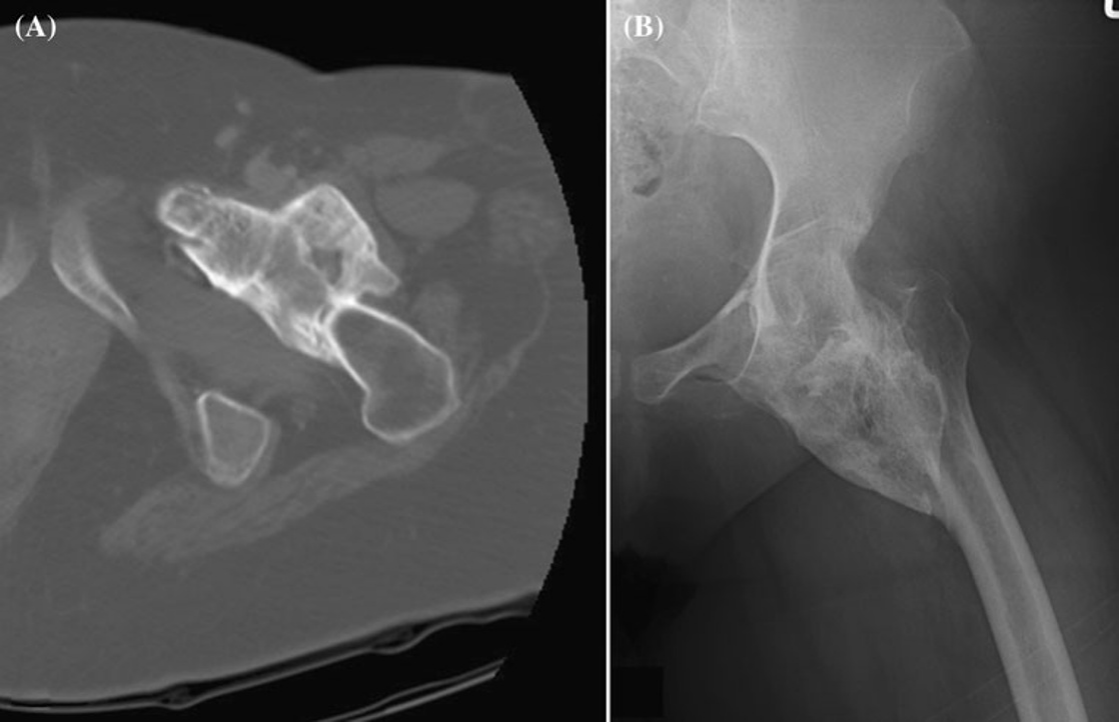
**a** Axial computed tomography scan and **b** anteroposterior radiograph of the left hip of a 49-year-old woman with anteromedial neurogenic HO of the hip after spinal cord injury (patient 10; type 3a)

**Fig. 5:**
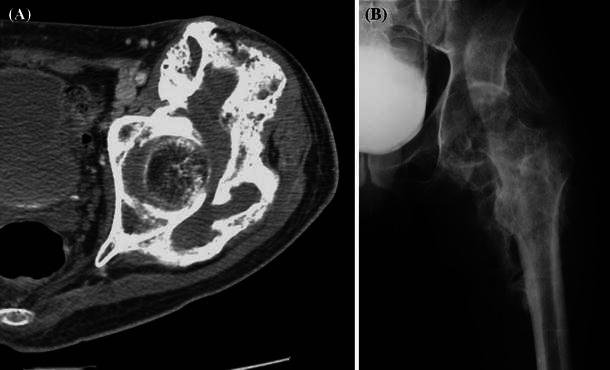
**a** Axial computed tomography scan and **b** anteroposterior radiograph of the left hip of a 43-year-old man with circumferential neurogenic HO around the hip after spinal cord injury (patient 23; type 4a)

We distinguished four types of neurogenic HO (Fig. 1): type 1 is characterized by anterior (Fig. 2), type 2 by posterior (Fig. 3), type 3 by anteromedial (Fig. 4), and type 4 by circumferential heterotopic bone formation (Fig. 5). A subtype was added to each type according to the etiology of the neurological injury: a, spinal cord injury; b, brain injury (Tables 1, 3). In all patients/hips, the classification proposed herein was straightforward and easy to assign. Preoperative planning facilitated the surgical excision of the heterotopic bone by choosing the appropriate surgical approach according to the anatomical location (type) of neurogenic HO; the anterior approach to the hip was used for anterior and anteromedial neurogenic HO (types 1 and 3), the posterior approach for posterior neurogenic HO (type 2), and a single-stage combined anterior and posterior approach for circumferential neurogenic HO (type 4).

Pre-treatment clinical examination showed a reduction in the range of motion and an inability to achieve a standing position in all patients, an inability to achieve a sitting position in 22 patients, hip ankylosis in 7 patients/14 hips, and hip pain in 1 patient (patient 1). According to the Brooker classification [1], all patients/hips were classified as class III or IV, which corresponds to hip ankylosis; however, only 7 patients/14 hips presented clinical or imaging evidence of ankylosis (Table 1). The mean hip flexion before treatment was 25° (range, 0–80°), extension 2.5° (range, 0–30°), internal rotation 5.5° (range, 0–10°), external rotation 6° (range, 0–20°), abduction 6° (range, 0–20°), and adduction 3° (range, 0–15°). After treatment, no patient had ankylosis of the hip joint, all patients were able to sit, and the mean range of hip motion significantly improved (*p* = 0.001); the mean hip flexion after treatment was 79° (range, 30–100°), extension 17° (range, 10–30°), internal rotation 25° (range, 5–35°), external rotation 23° (range, 5–30°), abduction 25° (range, 5–40°), and adduction 17° (range, 5–25°). Improvement was statistically significant for all motions (flexion, *p* = 0.035; extension, *p* = 0.030; internal rotation, *p* = 0.020; external rotation, *p* = 0.030; abduction, *p* = 0.030; adduction, *p* = 0.020), regardless of the type of neurogenic HO (*p* = 0.460).

Our classification also allowed for the estimation of blood loss,transfusion requirements, and recurrence of neurogenic HO. Overall, blood transfusion was necessary in 14 patients/20 hip operations. Although blood loss can be related to many factors, blood loss and transfusion requirements were statistically significantly higher in patients with type 3 and 4 compared to patients with type 1 and 2 neurogenic HO (chi-square test, *p* = 0.040). Overall, clinical and imaging recurrence of neurogenic HO was observed at 2 years in 5 patients/7 hips [21 %; patients 4, 6, 7 (bilateral), 10 (bilateral), and 17]; there were 4 patients/6 hips with neurogenic HO after brain injury (subtype b), and 1 patient/hip after spinal cord injury (subtype a). Recurrence of neurogenic HO was statistically significantly higher in patients with brain injury (Student’s *t* test, *p* = 0.040). Although recurrence was higher in patients with type 3 neurogenic HO, a statistically significant difference between the anatomical location of neurogenic HO and recurrence was not observed (Student’s *t* test, *p* = 0.198).

## Discussion

Neurogenic HO is a frequent complication in patients with central nervous system injury, and a potential cause of increased morbidity from complications resulting from immobilization [2, 11–14, 16, 23]. However, neurogenic HO is less well studied than the other HO types, and classifications for neurogenic HO are lacking [2, 11–14]; most of the data reported relate to patients with post-traumatic HO after total hip arthroplasty [17–19]. Therefore, we performed this study to propose a classification for neurogenic HO, to compare that classification with the Brooker classification [1], and to validate this classification in a clinical series of patients treated with combined surgical excision, indomethacin, and postoperative radiation therapy. The classification proposed herein distinguishes 4 types of neurogenic HO (types 1–4) according to the location of heterotopic bone formation around the hip joint, and 2 subtypes (a and b) according to the etiology of the neurological injury. Our results showed that the present classification can be useful for the management of neurogenic HO patients. It provides for preoperative planning of the surgical approach according to the anatomical location of the neurogenic HO, and permits an estimation of the prognosis regarding blood loss, transfusion requirements, and recurrence of the neurogenic HO.

We see four limitations in this study. First, the sample size is small; however, the lack of a classification for the specific HO type supports this study. Second, we did not use three-dimensional CT scan for the preoperative evaluation of HO. In this series and our practice, we use CT scan for the preoperative evaluation of HO patients, and axial CT scan views to classify HO and to indicate areas that should be avoided or carefully removed at surgery. Computed tomography scans may identify a low-density material in the soft tissue adjacent to areas of ectopic ossification that are postulated to be immature unossified connective tissue, the violation of which may be responsible for the serious intraoperative bleeding frequently experienced during the resection of HO [11]. Compared to three-dimensional CT reconstruction, axial CT scan is widely available and more easily read in clinical practice by most surgeons; also, in our opinion, it provides all of the information needed for preoperative planning. Third, we did not perform a volumetric quantification of the heterotopic bone, and did not include this volume in our classification criteria. We based our classification on the anatomical compartment involved by the neurogenic HO and not on its volume because we believe that the volume of HO is only related to the reduction in the range of motion or ankylosis and ease of surgical excision, not to the choice of surgical approach or the outcome of neurogenic HO. Moreover, including the volume of HO as a criterion would have made the classification more complex. Fourth, blood loss from surgical excision of the heterotopic bone can be related to many factors, and is not validated for this study’s purpose. However, we measured blood loss and transfusion requirements in order to provide a prognostic factor for the surgical treatment of each type of neurogenic HO.

A classification should meet certain criteria to be valuable and widely accepted. These should include ease of understanding, an ability to be easily recalled, consideration of the anatomy, an understanding of the mechanism of injury, the proposal of therapeutic guidelines according to the specific types, and the provision of useful information regarding the prognosis of the various types. We believe that the classification proposed herein addresses all of the above. The advantages of the Brooker classification are that it is based solely upon anteroposterior radiographs of the hip, and so it is a relatively simple and valid measurement that appears to correlate well with the clinical picture of overall hip function [24]. However, it does not address the anatomical compartment involved by HO, and does not correlate with the extent of HO into anatomical compartments, it cannot guide the surgical treatment or estimate prognosis, and it does not consider the etiology of the neurological injury that led to HO. Other methods have also been reported for the classification of HO in patients with post-traumatic HO and HO following hip arthroplasty [20–22]. These methods were based on the anteroposterior radiographic view of the hip, and classified HO according to the location around the femoral neck, without detailed anatomical localization. Some authors attempted to classify a central and lateral HO with respect to an imaginary borderline from the greater trochanter to the lateral edge of the acetabulum [25], or to divide the space around the femoral neck into thirds (central, lateral and medial) [26]; these classifications have not been widely accepted because of the complexity and difficulty involved in classifying HO into nonanatomical (imaginary) areas around the hip joint.

In the present classification, we distinguished 4 types (types 1–4) of neurogenic HO based on the anatomical compartments involved by the heterotopic bone. Since the etiology of neurogenic HO was found to be a statistically significant predictor for recurrence of HO, we added 2 subtypes (subtypes a and b) based on the etiology of neurological injury. The use of this classification made preoperative planning of the appropriate surgical approach rather straightforward. In the most common cases with anterior and/or medial HO (types 1 and 3), the anterior approach to the hip should be used; in cases of posterior HO (type 2), the posterior approach to the hip should be used; and in cases of circumferential HO (type 4), a combined anterior and lateral approach is recommended. Post-treatment improvement in the range of motion of the hip was significant in all cases, regardless of the type of neurogenic HO; therefore, the presence of ankylosis was not included in the criteria of our classification—it was only recorded to evaluate the effect of treatment. Additionally, HO that appears to be bridging according to the Brooker classification may actually be located either anterior or posterior to the hip, and thus may not cause significant loss of range of motion [21, 22]. This was also observed in the present clinical series; although according to the Brooker classification all of the patients/hips were classified as class III or IV, meaning hip ankylosis, clinical and imaging evidence of ankylosis was observed in only 7 patients/14 hips. The location (type) of neurogenic HO may also provide an estimate of blood loss and transfusion requirements. In the present study, blood loss and transfusion requirements were higher for patients with anteromedial (type 3) and circumferential (type 4) neurogenic HO. This may be explained by the fact that anteromedial or circumferential lesions and lesions in proximity to major vessels are more difficult to excise. A classification should also address the prognosis of a disease. Recurrence rates of neurogenic HO ranging from 17 to 58 % have been reported [10, 11, 13, 27]. In the present study, the recurrence rate of neurogenic HO was 21 % (5 patients/7 hips) at 2 years. The etiology of the neurological injury was found to be a significant prognostic factor for recurrence; recurrence was 6 times more common after brain injury (subtype b) compared to spinal cord injury (subtype a). We explain this by the fact that spinal cord injury patients may have a better performance status and selective motor control in the extremity, and can more easily achieve a better functional outcome [27–29]. The anatomical location of neurogenic HO was not found to be a significant prognostic factor for recurrence.

In conclusion, the management of neurogenic HO patients is challenging. A new classification specifically designed for this disorder is necessary. Since the trauma of surgery may actually aggravate the condition, adequate classification, preoperative planning, and combined treatment are beneficial.
